# Development and validation of a prognostic scoring system for cognitive decline in adults aged 50 and older in China

**DOI:** 10.3389/fnagi.2025.1664943

**Published:** 2025-09-23

**Authors:** Ningjie Zhang, Linglei Meng, Zhendong Qian, Ying Jin, Hua Yan

**Affiliations:** ^1^Department of General Practice, Ruijin Hospital, Shanghai Jiao Tong University School of Medicine, Shanghai, China; ^2^Department of General Practice, Huangpu District Dapuqiao Community Health Center, Shanghai, China; ^3^Department of Neurology, Shanghai Jiangong Hospital, Shanghai, China

**Keywords:** cognitive impairment, China, prognosis, scoring system, aging population

## Abstract

**Background:**

With the gradual aging of the global population, early identification and intervention are crucial for mitigating the negative impact of cognitive decline on patients and the healthcare system. This study aimed to develop and validate a prognostic scoring system for predicting cognitive decline in adults aged 50 and over in Shanghai, China.

**Methods:**

This community-based longitudinal study included 1,032 participants aged 50 and older with normal cognitive function at baseline. Of them, 986 participants were followed up for 2 years. Complete data from 864 individuals were randomly divided into derivation (*n* = 686) and validation (*n* = 178) cohorts and used to generate a prognostic scoring model. Sociodemographic and behavioral characteristics, comorbidities, and biochemical factors were collected from all participants. The least absolute shrinkage and selection operator (LASSO) logistic regression method was used to identify significant predictors. A multivariable logistic regression model was developed and validated using derivation and validation cohorts.

**Results:**

Of the thirteen variables initially selected, nine (age, gender, smoking, tea drinking history, hypertension, diabetes mellitus, coronary artery disease (CAD), hyperlipidemia, cerebral hemorrhage, and decline in daily function) were included in the final model. The nomogram-based scoring system showed moderate discriminatory power, with the area under the curve (AUC) of 0.65 and 0.67 in the training and validation sets, respectively, and good calibration.

**Conclusion:**

The developed prognostic scoring system provides a practical tool for predicting cognitive decline among adults aged 50 and older in Shanghai, China. The moderate discriminatory power and good calibration suggest that the model can effectively guide early interventions. Future research should validate the model in diverse populations and explore additional risk factors to enhance its predictive accuracy.

## Introduction

Cognitive decline is a pressing public health issue, particularly in China, where the aging population is expanding at an unprecedented rate (Lunenfeld and Stratton, 2013; Bai and Lei, 2020). Cognitive disorders may range from mild cognitive impairment (MCI) to severe dementia and have far-reaching implications not only for affected individuals but also for their families, caregivers, and the healthcare system in general (Aerts et al., 2017). With the continuous aging of the population, the prevalence of cognitive decline is currently on the rise (Prince et al., 2015; Bhatti et al., 2019). Patients with MCI are at a greater risk of developing Alzheimer’s disease (AD) or related dementia (Campbell et al., 2013). Recent advancements in the treatment of early AD with agents such as lecanemab, a humanized IgG1 monoclonal antibody that binds with high affinity to Aβ soluble protofibrils, further emphasize the need for early detection of MCI or preclinical AD stages. Such early screening, coupled with the timely initiation of pharmacological treatments, is crucial to mitigating the impact of the condition (Sabbagh et al., 2020).

Cognitive decline may be influenced by an interplay of various risk factors (Rao et al., 2023), including sociodemographic characteristics (age and gender), history of smoking and alcohol consumption, and various comorbidities such as hypertension, diabetes, dyslipidemia, etc (Okusaga et al., 2013; Han et al., 2023). Furthermore, the incidence of cognitive impairment accelerates from the sixth decade (Prince et al., 2024). At the same time, the major modifiable risk factors (e.g., hypertension, diabetes, dyslipidemia, obesity, smoking, depression, hearing loss, physical inactivity) are highly prevalent and actionable in late midlife (Rolandi et al., 2020), a window during which targeted management and closer follow-up can realistically alter next-visit risks. Therefore, developing a prognostic scoring system for this particular age group (adults aged 50 years and above) is crucial and will align with local screening and chronic disease management programs that typically begin in this age range.

However, despite extensive research, predicting cognitive decline remains challenging because traditional methods often fail to account for the intricate interrelationships between these factors (Hampel et al., 2011; Salthouse, 2011), underscoring the need for a more sophisticated and tailored prognostic approach.

Most existing studies and models are based on Western populations, which may have different risk profiles, lifestyle factors, and healthcare environments than those in China. However, China’s socio-demographic and cultural context and specific health-related characteristics necessitate a localized approach to predicting and managing cognitive decline (Wang et al., 2020; Chen et al., 2022). Therefore, there is a growing understanding that there is a need for a prognostic model specifically designed to cater to the unique characteristics of China’s population (Bao et al., 2022; van der Flier et al., 2023). Recently, several dementia/cognitive-impairment risk models have been developed or validated in Chinese cohorts (Hu et al., 2021; Hu et al., 2022; Zhou et al., 2020). While these studies advance the field, important gaps remain for frontline use. Many models rely on assessments or biomarkers that are not routinely available in primary care, report limited or no external validation, provide incomplete calibration or clinical utility evaluation (e.g., decision curve analysis), or are anchored to follow-up structures that differ from those seen in real-world clinics. In addition, most prior models target time-to-event outcomes that require precise onset dates. In contrast, routine services in many Chinese settings ascertain impairment status at discrete visits, leaving the onset time interval censored. Therefore, formulating prediction as a fixed-horizon (“next-visit”) risk task using indicators commonly available at the index visit, and evaluating both discrimination and calibration with transparent and reproducible methods, is imperative to address this practice-relevant scenario.

This study aims to generate and validate a prognostic scoring model for cognitive decline in adults aged 50 and older admitted to a tertiary care center in China. Healthcare providers can use such tools to identify individuals at high risk and implement timely interventions.

## Methods

### Study design

This community-based longitudinal study included a retrospective data analysis on a cohort of middle-aged and older adults from the Shanghai Brain Health Foundation (SHBHF2016001). Initially, the cohort included 1,032 individuals ≥50 years old with normal cognitive function and no signs of MCI or dementia at the time of recruitment. The study targeted adults aged 50 years or older to reflect the rising incidence of cognitive impairment in late midlife and the high prevalence of modifiable risk factors that are actionable in routine care. The intended use of the model is risk stratification at the index visit to estimate the probability of meeting criteria for cognitive impairment at the subsequent scheduled assessment (fixed-horizon prediction), rather than estimating time-to-event.

Over the course of 2 years, 986 participants remained in the study, while 46 participants were lost to follow-up. Of them, 122 participants had missing information for the variables included in the least absolute shrinkage and selection operator (LASSO) model. Finally, 864 participants constituted the final sample for analysis.

This study was approved by the Ethics Committee of the Shanghai Mental Health Center (No. 2018-11R, Date: 2018-07-27). Data collection commenced following ethical approval and written informed consent from all participants.

### Data collection

Candidate predictors: types, measurement, units, and preprocessing. Candidate predictors were pre-specified from routinely available clinical information at the index visit and grouped as follows.

(a) Demographics and history. Age (years; continuous), sex (female/male), years of formal education (years), marital status (married/other), and self-reported physician diagnoses of hypertension, diabetes, dyslipidaemia, coronary heart disease, stroke/TIA, and depression (yes/no). Medication classes (antihypertensives, antidiabetics, statins, antidepressants, anticholinergics) were extracted from the medication list (yes/no for each class).

(b) Lifestyle factors. Smoking (never/former/current); alcohol intake (never/occasional/weekly/daily; converted to standard drinks/week); tea consumption (never/≤3 cups/week/≥4 cups/week); physical activity from the short-form IPAQ expressed as MET-minutes/week (continuous) and categorized (low/moderate/high) for sensitivity checks; sleep duration (hours/night; continuous).

(c) Vital signs and anthropometrics. Systolic and diastolic blood pressure (mmHg) measured using a calibrated automated sphygmomanometer after ≥5 min seated rest; two readings 1–2 min apart, averaged. Heart rate (beats/min) recorded simultaneously. Height (cm) via wall stadiometer (no shoes) and weight (kg) via digital scale (light clothing); body mass index (BMI, kg/m^2^) computed as weight/height^2^. Waist circumference measured midway between the lowest rib and the iliac crest (cm) at end-expiration.

(d) Cognitive and functional measures at index visit. Baseline global cognition by the Montreal Cognitive Assessment (MoCA) Chinese version (0–30). Basic and instrumental activities of daily living (ADL/IADL) recorded using validated Chinese instruments; summary scores used as continuous predictors (higher = greater impairment). Depressive symptoms screened with PHQ-9 (0–27). Hearing/vision difficulty recorded (none/mild/moderate/severe) by self-report of functional limitation.

(e) Laboratory/biochemical indicators. Venous blood was drawn after an overnight fast (8–12 h) and analyzed in the hospital central laboratory accredited under routine internal and external quality control procedures. Units and methods were as follows:

- Fasting plasma glucose (mmol/L; hexokinase method); HbA1c (%; HPLC, NGSP/DCCT-aligned).
- Lipid profile: total cholesterol, LDL-C, HDL-C, triglycerides (all mmol/L; enzymatic colorimetric assays).
- Serum creatinine (μmol/L; enzymatic); eGFR (mL/min/1.73 m^2^; CKD-EPI equation).
- ALT/AST (U/L; kinetic method).
- High-sensitivity C-reactive protein (hs-CRP) (mg/L; immunoturbidimetric); homocysteine (μmol/L; enzymatic cycling).
- Uric acid (μmol/L; uricase); TSH (mIU/L; chemiluminescent immunoassay); vitamin B12 (pmol/L; chemiluminescence).

Preprocessing and standardization. To ensure comparability, all laboratory values were harmonized to SI units. If any values were recorded in conventional units, they were converted using standard factors before analysis. Right-skewed biomarkers (e.g., triglycerides, hs-CRP, homocysteine) were natural-log transformed. Continuous predictors were then standardized (z-scores: mean 0, SD 1) using parameters estimated from the training set; the same parameters were applied to the validation set to avoid information leakage. Categorical variables were dummy-encoded (one-hot). Outliers were handled via winsorisation at the 1st/99th percentiles within the training data before standardization. Missing values (when present) were imputed within the resampling/training folds only using simple, predictor-wise imputation (median for continuous, mode for categorical) to avoid leakage; the trained imputation parameters were then applied to the corresponding validation folds. No outcome information was used in any preprocessing step.

Cognitive status at follow-up was assessed using the Montreal Cognitive Assessment (MoCA), with a conventional threshold of <26 indicating cognitive impairment. MoCA is a screening instrument (not a diagnostic gold standard); therefore, some misclassification is expected. Since any residual misclassification is likely non-differential with respect to baseline predictors, its primary effect would be to attenuate effect sizes and modestly reduce discrimination, yielding conservative performance estimates for our model. Follow-up data were collected over two years to assess the participants’ cognitive status (Nasreddine et al., 2005).

The development of cognitive decline was considered a dependent variable. Independent variables included sociodemographic factors (age, sex, education, marital status, family history of dementia), behavioral characteristics (smoking, alcohol use, tea consumption), BMI, self-reported comorbidities (decline in daily living activities, sleep problems, DM, HTN, CAD, cerebral hemorrhage, cerebral infarction, depression, TBI) and biochemical parameters such as complete blood count parameters, levels of ALT, AST, total bilirubin, total cholesterol, LDL, HDL and triglycerides.

### Statistical analysis

STATA software v.14.2 was used for the analysis. Continuous variables were summarized as means and standard deviations (SD), while categorical variables were presented as proportions. The dataset was divided into derivation and validation cohorts. A prognostic scoring system was developed to predict the risk of cognitive decline using the derivation cohort and subsequently validated internally with the validation cohort.

For the model development, univariable logistic regression analysis was performed using all variables. The entire dataset was randomly split into training and validation sets at an 8:2 ratio.

## Development of the model

### Variable selection

The LASSO regression was done to select the variables to obtain a subset of variables that minimizes the error for a group of quantitative response variables (Musoro et al., 2014).

### Feature selection and hyperparameter tuning

A L1-penalized logistic regression (LASSO) was used to perform simultaneous shrinkage and variable selection for fixed-horizon risk prediction. All continuous predictors were standardized to a mean of zero and a variance of one prior to modeling; categorical variables were dummy-encoded. The regularization parameter (λ) was chosen exclusively via stratified 10-fold cross-validation within the training data, optimizing binomial deviance. To favor parsimony, when applicable, the one-standard-error rule was adopted, selecting the most regularized model whose cross-validated deviance lay within one standard error of the minimum. No hypothesis testing or *p*-value thresholds were used to select predictors at any stage; the active set of variables was determined solely by the cross-validated L1 penalty (Musoro et al., 2014). Model coefficients were then refit on the full training set at the selected λ and evaluated.

### Finalizing model

The multivariable logistic regression was done based on the variables identified through LASSO regression. The final set of predictors was determined by LASSO with λ selected via stratified 10-fold cross-validation. All variables were presented as adjusted odds ratios (aORs) with 95% confidence intervals (CIs).

### Sample size and model complexity

To control overfitting, the pragmatic “events-per-parameter” (EPP) rule of thumb was followed, targeting at least 20 outcome events per model degree of freedom. The derivation (training) set comprised *n* = 686 participants. The observed 2-year incidence of cognitive decline in the overall cohort was ≈28.4% (95% CI: 25.4%–31.5%), implying approximately E ≈ 195 events in the derivation set (0.284 × 686). Under an EPP ≥ 20 criterion, the derivation set supports about E/20 ≈9–10 effective parameters. The final penalized model included 9 degrees of freedom (one per retained predictor), thus satisfying the EPP ≥ 20 target. In addition, LASSO with stratified 10-fold cross-validation was used to select λ and shrink coefficients, further mitigating optimism beyond the EPP safeguard.

### Construction of nomogram-scoring system

A nomogram-scoring system was constructed to present the model using the “*nomolog*” package in STATA. Nomogram generation and calculation of predicted probability in a logistic regression model involved the following steps:

i. Scores were generated using the logit regression coefficients;

ii. The possible scores (α_1_ x_*i*_) were plotted for each variable (X_1_._*N*_).

iii. The constant value (α_0_) was obtained.

iv. The probability of event (p) was calculated:


$$p=\frac{1}{1+e^{-\left(\alpha_{0}+T\,P\right)}}$$

Total points = TP = α_1_ X_1_+ α_2_ X_2_+…

### Discrimination and calibration of the model

Receiver Operating Characteristic (ROC) analysis was performed by using the predicted probability of the event (p) as the test variable and the final true outcome as the gold standard variable. The area under the curve (AUC) was generated to explain the discriminatory ability of the nomogram-based model. The analysis was re-run in the validation set.

The calibration curve with the observed frequency was plotted against the predicted probability of cognitive decline to check calibration and identify any considerable deviation from 450 lines of a perfect fit in the training and validation sets (Collins et al., 2015).

## Results

The baseline characteristics of study participants are summarized in Table 1. The average age of patients in the training and validation cohorts was 70.0 (7.4) and 70.6 (7.8) years, respectively. Both cohorts had a higher percentage of female patients. There was a higher prevalence of hypertension in the training and validation cohorts (67.1 and 74.7%, respectively). Of 864 patients, 246 (28.5%; 95% CI: 25.4–31.5%) experienced cognitive decline during the 2-year follow-up period. The dataset was randomly split into training and validation sets at a 8:2 ratio (686 in the training set and 178 in the validation set).

**TABLE 1:** Baseline characteristics of the study participants.

Characteristics	Training set, *n* (%) *N* = 686	Validation set, *n* (%) *N* = 178
**Age of the patient**		
Mean age ( ± SD)	70.0 (7.41)	70.55 (7.80)
**Gender of the patient**		
Male	238 (34.7)	64 (36.0)
Female	448 (65.3)	114 (64.0)
**Marital status**		
Never married	99 (14.5)	21 (11.8)
Currently married	568 (83.0)	152 (85.4)
Widowed/separated/divorced	17 (2.5)	5 (2.8)
**Educational qualification**		
No formal education	20 (2.9)	6 (3.4)
Primary	55 (8.0)	12 (6.7)
Secondary	186 (27.1)	53 (29.8)
Higher secondary	225 (32.8)	57 (32.0)
Graduate	104 (15.2)	26 (14.6)
Post-graduate	96 (14.0)	24 (13.5)
**Smoking**		
No	561 (81.8)	144 (80.9)
Yes	125 (18.2)	34 (19.1)
**Alcohol user**		
No	587 (85.6)	146 (82.0)
Yes	99 (14.4)	32 (18.0)
**Tea drinker**		
No	348 (50.7)	97 (54.5)
Yes	338 (49.3)	81 (45.5)
**Hypertension**		
Present	460 (67.1)	133 (74.7)
Absent	226 (32.9)	45 (25.3)
**Diabetes mellitus**		
Present	181 (26.4)	54 (30.3)
Absent	505 (73.6)	124 (69.7)
**Coronary artery disease**		
Present	123 (17.9)	42 (23.6)
Absent	563 (82.1)	136 (76.4)
**Hyperlipidemia**		
Present	304 (44.3)	74 (41.6)
Absent	382 (55.7)	104 (58.4)
**Cerebral hemorrhage**		
Present	18 (2.6)	5 (2.8)
Absent	668 (97.4)	173 (97.2)
**Cerebral infarction**		
Present	163 (23.8)	43 (24.2)
Absent	523 (76.2)	135 (75.8)
**Depression**		
Present	74 (10.8)	19 (10.7)
Absent	608 (89.2)	158 (89.3)
**Decline in daily function**		
Present	26 (3.8)	8 (4.5)
Absent	660 (96.2)	170 (95.5)
**Physiological and biochemical parameters**		
Body mass index (BMI in kg/m^2^) mean (±SD)	24.07 (3.54)	24.47 (3.42)
Red cell count (RBC) mean (±SD)	4.39 (0.61)	4.39 (0.67)
Blood platelets mean (±SD)	2.08 (0.64)	2.01 (0.56)
Mean platelet volume mean (±SD)	9.25 (1.07)	9.26 (1.16)
Lymphocyte count mean (±SD)	2.15 (0.74)	2.11 (0.70)
Hemoglobin concentration mean ( ± SD)	13.23 (1.8)	13.23 (2.02)
Alanine aminotransferase mean (±SD)	22.02 (11.3)	26.11 (16.65))
Total protein mean (±SD)	7.6 (0.45)	7.6 (4.57)
Aspartate aminotransferase mean (±SD)	23.15 (7.30)	25.72 (10.57)
HDL cholesterol mean (±SD)	1.37 (0.37)	1.37 (0.36)
LDL cholesterol mean (±SD)	3.09 (0.91)	2.99 (1.12)
Total cholesterol mean (±SD)	5.04 (1.02)	5.02 (1.20)
Triglycerides mean (±SD)	1.66 (1.00)	1.85 (1.58)

A total of 686 patients were included in the training set, of whom 190 (27.7%) had cognitive impairment and 496 (72.3%) did not. The overall mean age was 70.0 ± 7.41 years, with patients exhibiting cognitive impairment being slightly older (71.11 ± 7.54 years) than those without (69.56 ± 7.32 years). Females comprised 65.3% of the cohort, with a higher proportion among those with cognitive impairment (69.5%) compared to those without (63.7%). Regarding marital status, 83.0% were currently married, 14.5% were never married, and 2.5% were widowed, separated, or divorced. In terms of educational qualifications, the most common level was higher secondary (32.8%), followed by graduate (15.2%) and post-graduate (14.0%). Most patients were non-smokers (81.8%) and non-alcohol users (85.6%); however, tea consumption was reported by 49.3% of the total sample, with a lower proportion among those with cognitive impairment (40.0%) compared to those without (52.8%). Comorbid conditions were common, with hypertension present in 67.1%, diabetes mellitus in 26.4%, coronary artery disease in 17.9%, and hyperlipidemia in 44.3% of patients. Cerebral hemorrhage and infarction were reported in 2.6 and 23.8% of patients, respectively, while depression was noted in 10.8% and a decline in daily function in 3.8%. Physiologically, the overall body mass index was 24.07 ± 3.54 kg/m^2^, the red cell count 4.39 ± 0.61, and hemoglobin concentration 13.23 ± 1.80 g/dL. Biochemical parameters were as follows: alanine aminotransferase (22.02 ± 11.30 U/L), aspartate aminotransferase (23.15 ± 7.30 U/L), HDL cholesterol (1.37 ± 0.37 mmol/L), LDL cholesterol (3.09 ± 0.91 mmol/L), total cholesterol (5.04 ± 1.02 mmol/L), and triglycerides (1.66 ± 1.00 mmol/L).

### Development of a nomogram-based model

**FIGURE 1:**
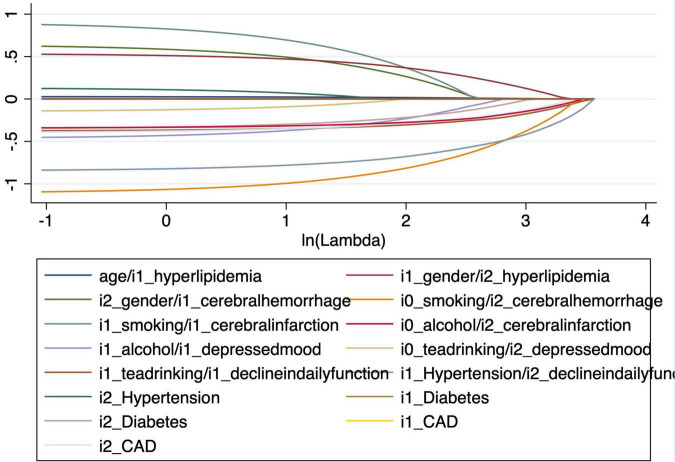
LASSO (Least Absolute Shrinkage and Selection Operator) coefficient profile plots for variables associated with cognitive decline. The x-axis represents the log-transformed lambda values, and the y-axis shows the coefficients of predictors included in the model. Variables retained as significant predictors are shown with nonzero coefficients.

Based on the lambda value selected by EBIC (Figure 1), 13 variables (age, gender, smoking, alcohol, tea drinking history, hypertension, diabetes mellitus, CAD, hyperlipidemia, cerebral hemorrhage, cerebral infarction, depression, decline in daily function) were chosen for the nomogram-based model. Multivariable logistic regression was then performed using the set of selected variables. As shown in Table 2, the results of the analysis showed that age (OR:1.03, 95% CI: 1.01–1.05; *P* = 0.020), female gender (OR: 2.09, 95% CI: 1.25–3.51; *P* = 0.005), smoking (OR: 2.31 95% CI: 1.27–4.20; *P* = 0.006), history of tea consumption (OR:1.55, 95% CI: 1.07–2.24, *P* = 0.020), absence of hyperlipidemia (OR:0.60, 95% CI: 0.41–0.86; *P* = 0.005), cerebral hemorrhage (OR:3.03, 95% CI: 1.12–5.68; *P* = 0.029), and decline in the daily function (OR: 2.45, 95% CI: 1.06–5.68; *P* = 0.036) were all risk factors that may predict cognitive decline, and were selected for the final nomogram-based model (Table 2).

**TABLE 2:** Multivariable logistic regression for the predictors of cognitive decline based on training set (*N* = 686).

Characteristics	Adjusted OR	S.E.	95% CI	*P*-value
Age of the patient	1.03	0.013	1.01–1.05	0.020*
**Gender**				
Females	2.09	0.55	1.25–3.51	0.005*
Males	Ref	–	–	-
**Smoking**				
Non-smoker	Ref	–	–	-
Smoker	2.31	0.71	1.27–4.20	0.006*
**History of tea consumption**				
Regular drinker	Ref	–	–	-
Non-drinker	1.55	0.29	1.07–2.24	0.020*
**Diabetes mellitus**				
Absent	Ref	–	–	-
Present	1.42	0.28	0.97–2.10	0.074
**Coronary artery disease**				
Absent	Ref	–	–	-
Present	1.51	0.33	0.98–2.33	0.064
**Hyperlipidemia**				
Present	Ref	–	–	-
Absent	0.60	0.11	0.41–0.86	0.005*
**Cerebral hemorrhage**				
Absent	Ref	–	–	-
Present	3.03	1.53	1.12–5.68	0.029*
**Decline in the daily function**				
Absent	Ref	–	–	-
Present	2.45	1.05	1.06–5.68	0.036*

OR: odds ratio; S.E: standard error; CI: confidence interval; Ref: reference category.

*p-value < 0.05 i.e., statistically significant.

### Nomogram-scoring system

**FIGURE 2:**
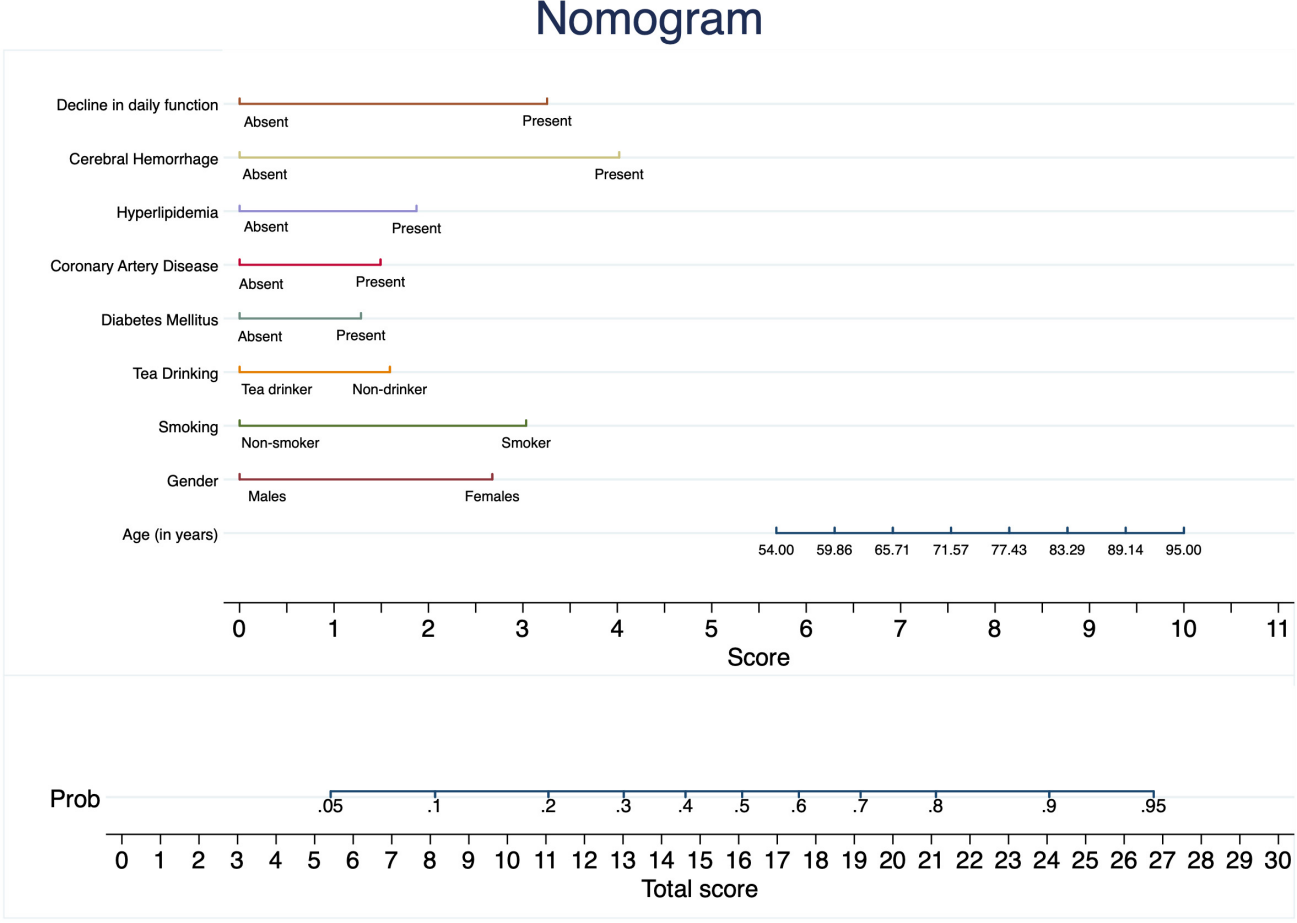
Nomogram scoring system developed to predict the risk of cognitive decline among older adults in Shanghai, China. Each predictor corresponds to a score based on its presence or value, with the total score providing the estimated probability of cognitive decline.

The final model was presented as a scoring system (Figure 2). The selected variables in the nomogram-based model were arranged one by one on a horizontal plane in the scoring system, ranging from 0 to 10 at the bottom. The total score ranged from 0 to 29.3, and the scores of individual variables are provided in Table 3.

**TABLE 3:** Scoring system for prediction of cognitive decline amongst older adults.

Characteristics	Range or status	Score (points)
Age of the patient (in years)	≥95	10.0
	89–94	9.4
	83–88	8.8
	77–82	8.2
	72–76	7.5
	66–71	6.9
	60–65	6.3
	54–59	5.7
Cerebral hemorrhage	Present	4.0
	Absent	0.0
Smoking	Smoker	3.0
	Non-smoker	0.0
Decline in daily function	Present	3.3
	Absent	0.0
Gender	Females	2.7
	Males	0.0
Coronary artery disease	Present	1.5
	Absent	0.0
Tea drinking	Non-drinker	1.6
	Tea drinker	0.0
Hyperlipidemia	Present	1.9
	Absent	0.0
Diabetes mellitus	Present	1.3
	Absent	0.0

The nomogram calculates the total score for each individual by summing the points assigned to each risk factor, and the corresponding probability of cognitive decline can be determined based on the total score. For example, a total score of 15 points corresponds to a probability of approximately 0.50 (50%) for cognitive decline. Higher total scores indicate a higher likelihood of cognitive decline, allowing clinicians to interpret and apply the prognostic model in clinical settings easily.

### Discrimination and calibration

**FIGURE 3:**
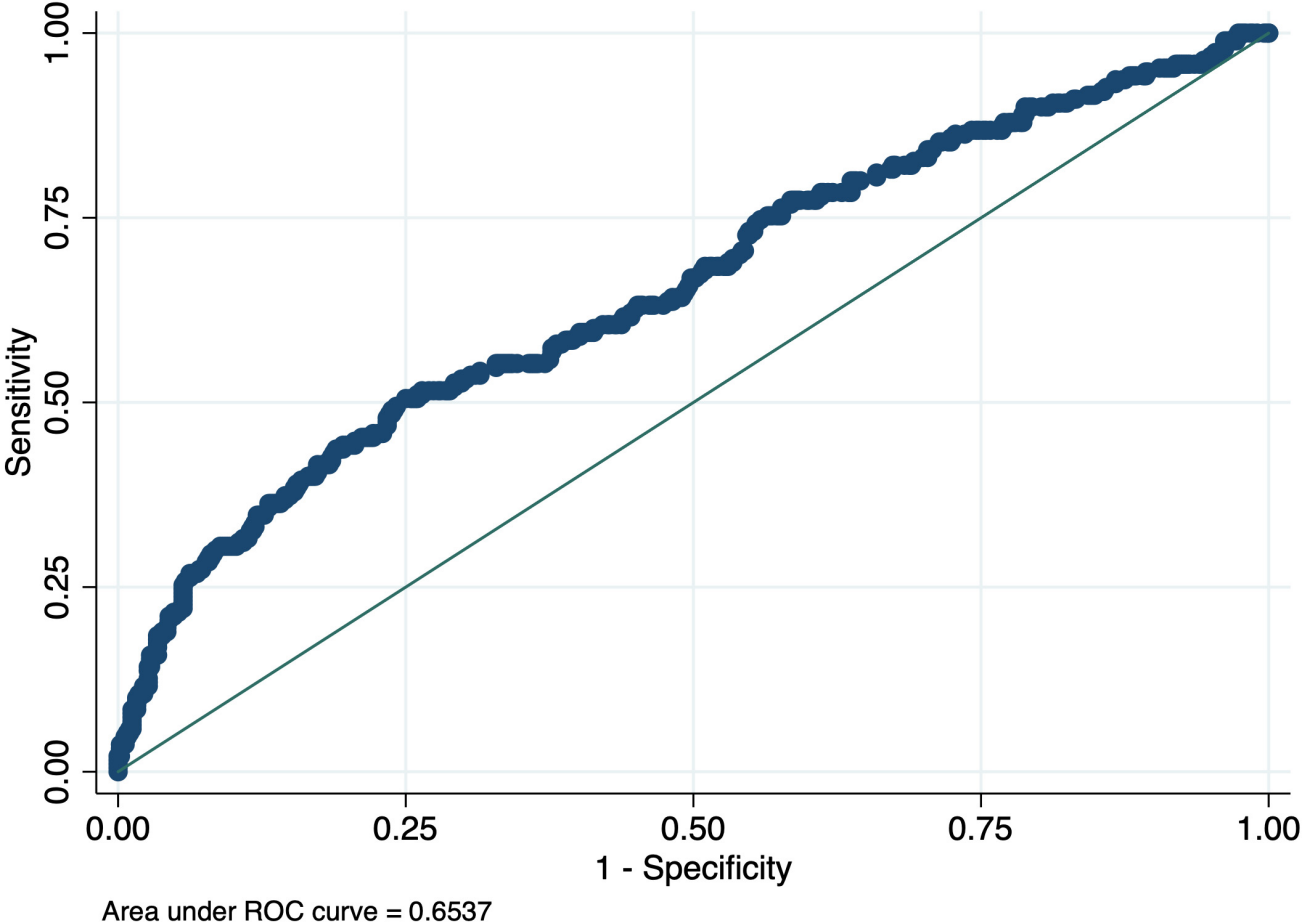
Receiver Operating Characteristic (ROC) curve for the derivation cohort. The curve illustrates the trade-off between sensitivity and specificity of the prediction model, with an area under the curve (AUC) of 0.65 indicating moderate discriminatory power.

**FIGURE 4:**
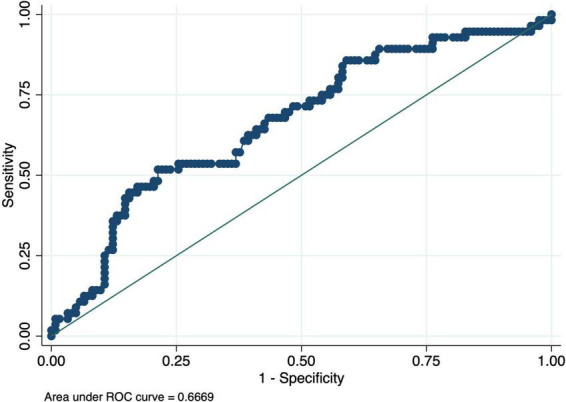
Receiver Operating Characteristic (ROC) curve for the validation cohort. The curve demonstrates the performance of the prediction model on the validation dataset, with an area under the curve (AUC) of 0.67, reflecting moderate accuracy.

**FIGURE 5:**
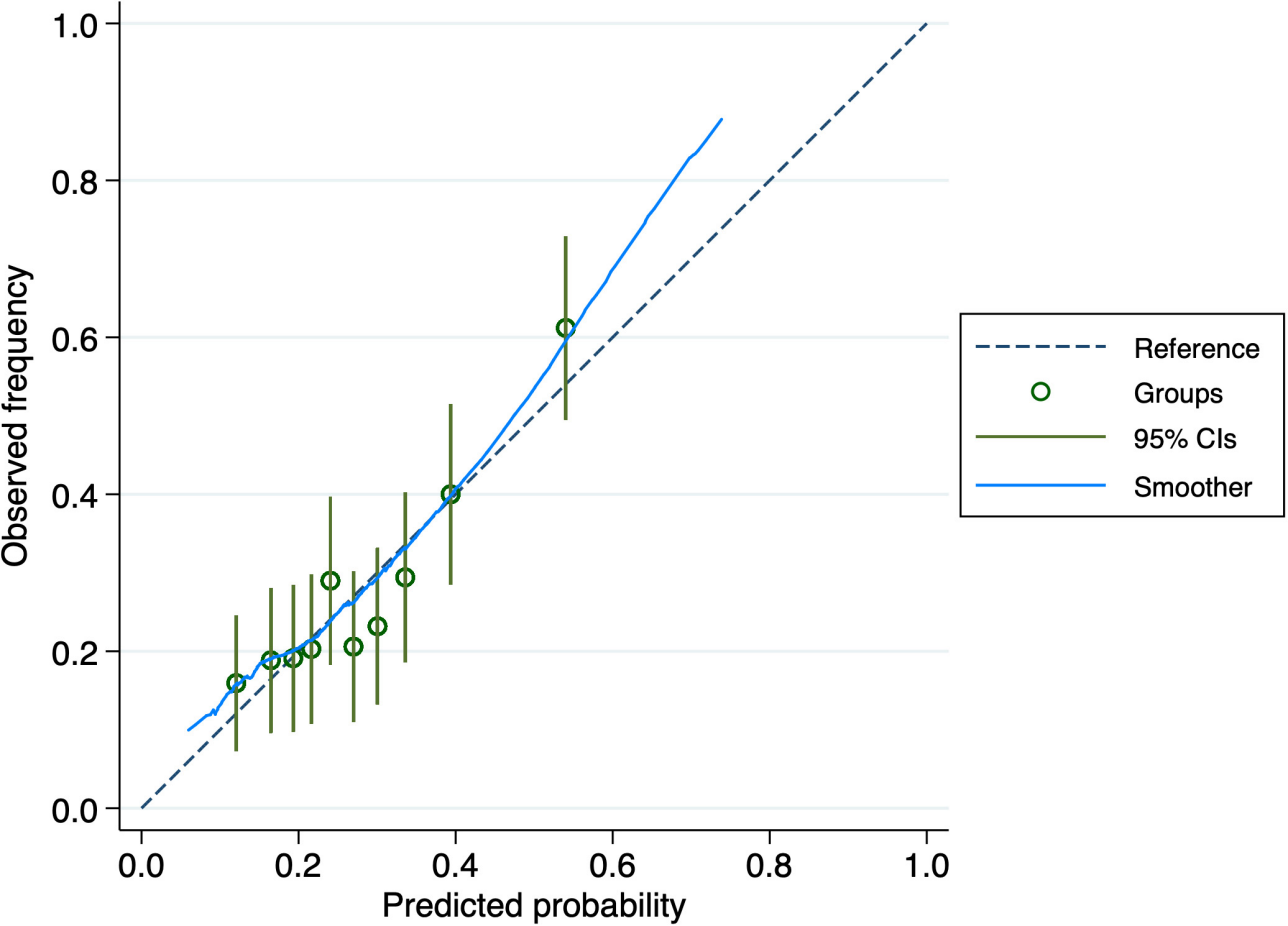
Calibration curve for the derivation cohort. The plot compares observed frequencies of cognitive decline (y-axis) with predicted probabilities (x-axis), with the dashed line representing perfect calibration. The 95% confidence intervals (vertical bars) indicate variability within each group.

**FIGURE 6:**
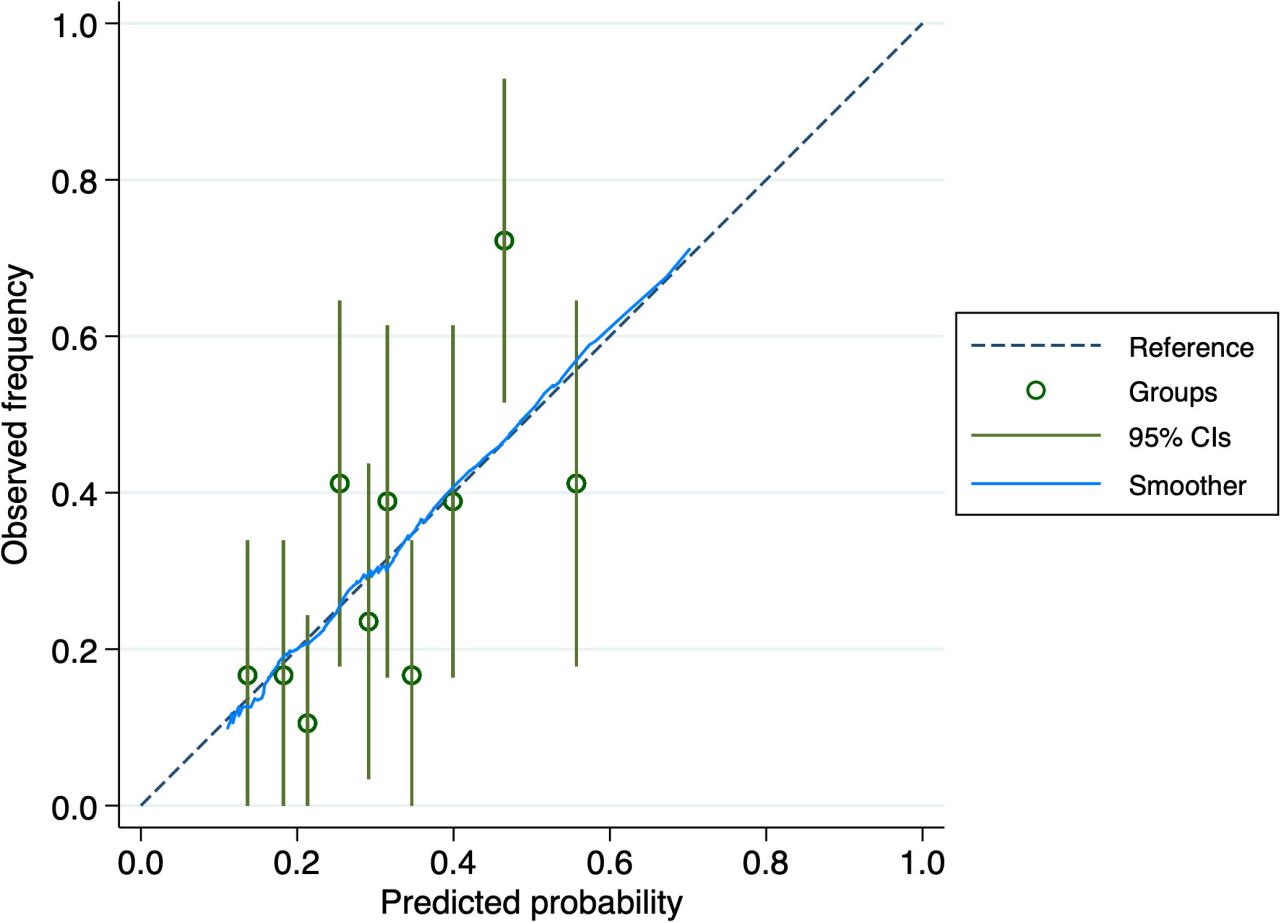
Calibration curve for the validation cohort. The plot assesses the agreement between observed and predicted probabilities of cognitive decline. The dashed line represents perfect calibration, and the blue smoothed curve shows the actual calibration performance with 95% confidence intervals.

The nomogram-based model’s discriminatory power in the training set was 0.65 (95% CI: 0.61–0.70) (Figure 3). Similar discriminatory power was detected for the validation set (0.67; 95% CI: 0.58–0.75) (Figure 4). Figures 5, 6 illustrate the calibration plot for predicted probabilities versus observed outcomes, demonstrating the model’s predictive performance in both the derivation set and the validation set. The points represent grouped predicted probabilities, along with their corresponding observed frequencies, and 95% CIs (vertical bars). The blue smoother line closely follows the diagonal reference line, indicating a good agreement between predictions and observed outcomes. This suggests that the model demonstrates acceptable calibration with no significant deviations across the probability range.

## Discussion

The current study aimed to develop and validate a prognostic scoring system to predict cognitive decline in individuals aged 50 and older in Shanghai, China. The final model included nine variables: age, gender, smoking, tea-drinking history, hypertension, diabetes mellitus, CAD, hyperlipidemia, cerebral hemorrhage, and decline in daily function. The model demonstrated moderate discriminatory power and good calibration in the training and validation sets, indicating its robustness and potential utility in clinical and community settings. The model and findings were consistent with those of models developed in the existing literature (Hu et al., 2021; Hu et al., 2022; Zhou et al., 2020).

The results showed that age and gender are significant predictors of cognitive decline, consistent with the previous research. Numerous studies have highlighted the strong association between aging and cognitive deterioration and identified age ss the most significant predictor (Dufouil et al., 1996; Schönknecht et al., 2005; Ma et al., 2020; Yang et al., 2023). The gender differences observed in this study also reflect the findings from other research, indicating that females are at a higher risk of cognitive decline, possibly due to hormonal differences and longer life expectancy (Sohn et al., 2018; Rahman et al., 2019; Levine et al., 2021; Verhagen et al., 2022). These associations may be explained by several mechanisms and existing theoretical frameworks. Age emerged as the most significant predictor of cognitive decline, a finding consistent with the natural aging process. Aging is associated with a cumulative burden of oxidative stress, inflammatory processes, and cellular senescence, all contributing to neuronal damage and cognitive impairment (Liguori et al., 2018). The longer life expectancy of women, coupled with the hormonal changes that occur post-menopause, may explain why gender also emerged as a significant predictor, with females showing a higher risk of cognitive decline (Han et al., 2023).

The impact of various behavioral factors on cognitive health has been extensively studied. This study demonstrated a higher risk of cognitive decline among smokers, which is consistent with previous research (Anstey et al., 2007; Peters et al., 2008; Sabia et al., 2012). Nicotine and other harmful substances in cigarettes are known to cause vascular damage and reduce cerebral blood flow, leading to neurodegeneration (Mazzone et al., 2010). Smoking also increases oxidative stress and inflammation, which can accelerate cognitive decline (Peters et al., 2008). This aligns with existing literature that consistently finds a strong association between smoking and various forms of cognitive impairment (Ford et al., 1996; Liu et al., 2017).

The literature has been less consistent on the impact of tea drinking on cognitive health. Several studies suggested a protective effect due to the antioxidants present in tea (Ng et al., 2008; Sargent et al., 2020; Wei et al., 2023). In agreement with these observations, the findings of this study indicate that non-tea drinkers are at a slightly higher risk of cognitive decline.

The role of comorbid conditions such as hypertension, diabetes, CAD, and hyperlipidemia in cognitive decline has been well-documented. These conditions affect vascular health, which is crucial for maintaining cognitive function (Alagiakrishnan et al., 2006; Duron and Hanon, 2008; Cohen et al., 2009). This study supports such associations, showing that hypertension, diabetes, CAD, and hyperlipidemia are all significantly associated with cognitive deterioration, further highlighting the importance of managing cardiovascular health to prevent mental decline. All these conditions are known to impair blood flow to the brain, leading to ischemia (Alagiakrishnan et al., 2006; Duron and Hanon, 2008; Cohen et al., 2009). Hypertension and diabetes can cause microvascular damage, which affects the brain’s white matter and leads to cognitive deficits (van Sloten et al., 2020). The relation between hyperlipidemia and cognitive decline can be due to the formation of atherosclerotic plaques, which limit blood flow to the brain (Duong et al., 2021). The significant impact of cerebral hemorrhage on cognitive decline observed in our study is also in line with previous research (Potter et al., 2021; El Husseini et al., 2023). Cerebral hemorrhage directly damages brain tissue and disrupts normal brain function (Ungvari et al., 2017). The presence of cerebral hemorrhage as a significant predictor in this model highlights the severe and immediate effects of such vascular events on cognition.

Contrary to some studies that did not find a link between depression and cognitive decline, the initial model in this study included depression as a predictor (Kim, 2022). However, it was excluded from the final model due to a lack of statistical significance, suggesting that while depression may be related to cognitive decline, its impact may not be as strong as other factors in our population.

The decline in daily function that was more frequently reported in older patients with cognitive decline in the present cohort may be considered both a symptom and a predictor of cognitive decline (Cipriani et al., 2020; Dubbelman et al., 2020; Oveisgharan et al., 2024). As cognitive abilities diminish, individuals are less able to perform daily activities, and this reduction in daily function can further exacerbate cognitive deterioration through reduced mental and physical activity.

### Strengths and limitations of the study

The main goal of the study was not to replace existing models but to provide a parsimonious, deployable tool tailored to routine Chinese clinical workflows where outcomes are assessed at discrete visits and detailed onset times are unavailable. By specifying a fixed-horizon risk target, relying on readily obtainable predictors, and reporting calibration and simple decision-support thresholds, the study complemented prior work and enabled head-to-head external validation against existing Chinese models in future studies.

The current study involved a substantial number of participants, which increases the statistical power and generalizability of our results. A two-year period allowed for capturing changes in cognitive function over time, providing a more dynamic understanding of cognitive decline. A wide range of acquired sociodemographic, behavioral, comorbid, and biochemical data allowed for a thorough analysis of multiple risk factors. Using LASSO logistic regression and EBIC for model selection ensured that the most relevant predictors were identified without overfitting. The model was internally validated with an external cohort, adding robustness. The developed nomogram-based scoring system provides a practical tool that can be readily used in clinical and community settings to identify patients at high risk of cognitive decline.

This study has some limitations. The main limitation is that the outcome was defined using a screening tool (MoCA < 26) rather than a clinical diagnosis. While MoCA has good sensitivity and acceptable specificity for detecting cognitive impairment, misclassification is still possible. Non-differential outcome misclassification would likely bias the results toward the null and slightly lower the AUC, meaning our estimates of discrimination are probably conservative. Future validation against confirmed clinical outcomes (and exploring education-adjusted thresholds) will help determine any effects on calibration and net benefit. Discrimination was modest (AUC around 0.65 in the derivation set and about 0.67 in the validation set), which may limit its use in standalone clinical decisions. This probably reflects our baseline-only predictor set and the use of a screening test to define cognitive impairment at follow-up, both of which can reduce the apparent ability to distinguish risks. The validation was internal to the available cohort and does not replace independent external validation across different sites and case-mixes. Therefore, generalizability remains uncertain. Predictors included only demographics, medical history, and routine blood measures to maximize feasibility and equity in primary care settings. The exclusion of APOE genotyping and neuroimaging likely limited the maximum discrimination and may partly explain the moderate AUC.

The study only included patients from a single community in Shanghai. Future studies should include participants from other regions or countries with different demographic and cultural characteristics to improve generalizability. Additionally, the study relied on the self-reporting of behavioral and comorbid conditions, which may have resulted in recall or social desirability bias, potentially affecting the accuracy of the information. Although the study is longitudinal, some variables were only measured at baseline, limiting the ability to capture changes in these factors over time and their impact on cognitive decline. The model’s discriminatory power was moderate (AUC around 0.65–0.67), suggesting that there may be other unmeasured factors influencing cognitive decline that were not captured in this study.

### Implications for clinical practice

Despite limitations, the reported findings have significant implications for clinical practice and public health policy. The developed prognostic scoring system provides a practical and reliable tool for healthcare providers to assess the risk of cognitive decline in middle-aged and older adults on time, enabling them to implement targeted interventions that delay or prevent the onset of cognitive decline. Healthcare providers can use the model to screen patients during routine visits, particularly focusing on older adults with multiple risk factors such as advanced age, smoking history, and cardiovascular comorbidities. Early intervention strategies, such as cognitive training, lifestyle modifications, and managing cardiovascular risk factors, can be more effectively applied to patients at high risk.

The reported predictions are intended to guide care planning, not to diagnose dementia. In practice, a “high-risk” classification can trigger proportionate actions that are already available in routine settings: (i) optimization of vascular and metabolic risk factors; (ii) review and reduction of anticholinergic burden and other potentially inappropriate medications; (iii) evaluation and correction of hearing and vision deficits; (iv) screening and management of depression, sleep problems, and social isolation; and (v) closer interval follow-up or referral for formal cognitive assessment when indicated. Framed this way, risk stratification supports targeted prevention and earlier evaluation beyond generic lifestyle advice, aligning with pragmatic resource allocation in busy clinics.

Additionally, the detailed scoring system in this study enables clinicians to design care plans tailored to each patient’s individual risk profile. For instance, a smoker with hypertension and diabetes may receive a tailored intervention plan that includes smoking cessation support, blood pressure control, and diabetes management. Personalized interventions can be more effective in mitigating risk factors and slowing the progression of cognitive decline.

Moreover, by identifying high-risk populations, health systems can prioritize resource allocation to areas and groups most in need, such as cognitive health programs and preventive services. This targeted approach can maximize the impact of available resources, improve population health outcomes, and reduce the burden on the healthcare system.

This study highlights the importance of lifestyle factors, including smoking cessation and cardiovascular health, in preventing cognitive decline. Public health campaigns can use these findings to educate the community about the importance of healthy living and its impact on cognitive health. Increasing public awareness can motivate individuals to adopt healthier behaviors and seek regular health check-ups.

While this study provides a robust framework for predicting cognitive decline, further research is necessary to enhance and expand upon these findings. Future studies should include diverse populations from different geographic regions and cultural backgrounds to validate and potentially refine the prognostic scoring system. This will ensure the model is generalizable and applicable to various populations, increasing its utility and effectiveness. Although this study followed participants for two years, longer follow-up periods are essential to capture the long-term progression of cognitive decline. Extended follow-up can provide deeper insights into the trajectory of cognitive changes and the impact of various risk factors over time. Further studies are needed to elucidate the underlying mechanisms linking the identified risk factors with cognitive decline. Understanding the biological and physiological pathways of cognitive decline can inform the development of more targeted and effective interventions. Integrating technology, such as mobile health applications and wearable devices, can facilitate the continuous monitoring of risk factors and cognitive function. Future research should explore the feasibility and effectiveness of using technology to enhance early detection and intervention strategies. Research should also focus on the impact of policy changes informed by our findings. Evaluating the effectiveness of public health interventions and resource allocation strategies based on the prognostic model can provide valuable feedback for continuous improvement.

## Conclusion

This study successfully developed and validated a practical prognostic scoring system for predicting cognitive decline among middle-aged and older adults in Shanghai, integrating key sociodemographic, behavioral, and comorbid factors. The model demonstrates moderate discriminatory power and good calibration, offering a valuable tool for early identification and intervention. Future research should focus on validating the model across diverse populations and exploring additional risk factors to enhance predictive accuracy.

## Data Availability

The raw data supporting the conclusions of this article will be made available by the authors, without undue reservation.
